# The effect of scleral buckling on accommodative amplitude

**DOI:** 10.1186/s40942-020-00218-z

**Published:** 2020-04-20

**Authors:** Nazanin Ebrahimiadib, Narges Hassanpoor, Mohammadreza Niyousha, Bobeck Seyed Modjtahedi

**Affiliations:** 1 Retina & Vitreous Service, Farabi Eye Hospital, Tehran University of medical sciences, Tehran, Iran; 2 Retina & Vitreous Service, Nikookari Eye Hospital, Tabriz University of medical sciences, Tabriz, Iran; 3 Eye Monitoring Center, Kaiser Permanente Southern California, 1011 Baldwin Park Blvd, Baldwin Park, CA 91706 USA; 4 Eye Research Center, Farabi Eye Hospital, Qazvin Square, Tehran, Iran

**Keywords:** Scleral expansion, Presbyopia, Accommodation, Buckling, Encircling band, Retinal detachment

## Abstract

**Purpose:**

To evaluate the effect of scleral buckling on accommodative amplitude.

**Design:**

Non-randomized, prospective, double masked clinical trial in which the fellow eye of patients undergoing scleral buckling served as a control.

**Methods:**

Patients who underwent scleral bucking for the management of retinal detachment in at least one eye were enrolled. Accommodative amplitude was measured monocularly 1 month and 3 months post operatively by two masked optometrists using a near-point “push” technique and minus-to-blur technique. Accommodative amplitude in eyes that underwent scleral buckle surgery were compared to their fellow eyes. Buckle type, buckle location, lens status and age were analyzed. Generalized Estimation Equations (GEE) were used to compare means and percentages between two groups.

**Results:**

Seventy-four eyes of 37 patients were included in the study. Median age was 44 years old (range: 31–67 years old) and 68.4% of patients were male (n = 24). Two patients required bilateral surgery. Thirty-six of 39 operated eyes (92.3%) were phakic and three were pseudophakic. In phakic eyes there was a significantly higher amplitude of accommodation in operated eyes compared to their fellow eyes at post-operative month one (0.99 diopters, p value = 0.002) and three (1.17 diopters, p value = 0.001). The difference in accommodative amplitude in post-operative eyes compared to control eyes did not reach statistical significance in pseudophakic eyes nor did it differ between those who had an encircling band and those with a segmental buckle at both one and 3 months after surgery (p value = 0.37 and 0.38, respectively). In those with a segmental buckle, inferior fixation resulted in a larger difference in accommodative amplitude compared to control eyes than any other location fixation. Age under 40 years old and better post-operative best corrected visual acuity (BCVA) both correlated with greater difference in accommodative amplitude compared to fellow eyes.

**Conclusion:**

Compared to fellow eyes not undergoing surgery, those eyes that underwent scleral buckling had a greater accommodative amplitude with larger differences correlating with better post-operative BCVA and younger age.

## Introduction

The mechanisms responsible for accommodation remain incompletely understood. Contraction of the ciliary muscles resulting in phakic lens steepening was proposed by Von Helmholz [1, 2] while pulling of the lens equator towards the sclera by equatorial zonuleshas also been theorized to induce accomodation [3–6] Attempts manipulate the sclera to treat presbyopia have evolved and are gaining increasing attention. These efforts include scleral expansion including with implantation of band segments of polymethylmethacrylate (PMMA) which Qazi et al. found resulted in modest increases in accommodative amplitude [1]. Scleral buckling similarly results in expansion of the sclera. Although myopic shifts following scleral buckling surgery are well characterized, less is known about the effect of this surgery on accommodation.

This study sought to characterize differences in accommodative amplitude following scleral buckling surgery in patients undergoing retinal detachment repair.

### Patients and methods

Patients who underwent scleral buckling surgery for retinal detachment repair in at least one eye between July 1, 2018 and June 1, 2019 were included in this prospective study. All patients underwent complete ophthalmic evaluations including manifest refraction post-operatively as well as detailed examinations of the anterior and posterior segments both pre- and post-operatively. Accommodative amplitude was measured monocularly in all patients by both near-point “push” technique and minus-to-blur technique at post-operative months one and three [1, 6]. Examinations were independently conducted by two masked optometrists. All patients discontinued cycloplegic drugs at least 14 days before assessment of accommodative amplitudes. After evaluation of inter-rater agreement level, the mean of both measurements of accommodative amplitude was used for analysis. Two patients underwent bilateral scleral buckling surgery due to bilateral retinal detachment. Fellow eyes of patients with unilateral surgeries (35 eyes) served as the control in this study. Due to including both eyes of bilateral cases in the “case” group, we have more study eyes than control eyes in this paper.

Patients were classified based on age, lens status, buckle type (encircling versus segmental), and buckle location in the case of segmental buckles.

Patients with a history of prior ocular surgeries (other than cataract extraction), prior ocular history other than refractive error, different phakic status in each eye, best corrected visual acuity (BCVA) less than 0.6 logMAR in the fellow eye, 1 month post-operative BCVA less than 1.0 logMAR in operated eye, failure of primary scleral buckle repair, history of diabetes mellitus and/or neurologic disease that can cause accommodation disability, inability to participate in subjective visual tests, or use of any topical or systemic medication that could impact accommodation within 2 weeks of measurements.

Scleral buckles were secured to the scleral using partial thickness passes of 5–0 mersilene sutures. The type of buckle and the decision between encircling versus segmental buckles were done at the discretion of the surgeon to achieve the best chance at retinal detachment repair. Buckle height was adjusted intraoperatively to achieve appropriate indentation as judged by the surgeon. Segmental buckles covered at least 180 degrees of the sclera in most cases.

Written informed consent was acquired by patients prior to enrollment to the study. The study adhered to the tenets of the Declaration of Helsinki and Institutional Review Board (IRB) approval was attained from the Tehran University of Medical Sciences.

Statistical analyses were performed with SPSS software (IBM SPSS Statistics for Windows, Version 22.0., Armonk, NY: IBM Corporation). Descriptive statistics were used to evaluate the distribution of the data. Continuous data with normal distribution were given as mean ± standard deviation or median. Both eyes of one subject in this paper were included which may cause errors using usual T test analyses considering the dependency between two eyes in one subject. Therefore, the other statistical analyses were carried out using STATA software version 14.0 (StataCorp, College Station, Texas, USA). Generalized estimation equations (GEE) were used to compare means and percentages between two groups. A p value of 0.05 or less was considered statistically significant.

### Results

Thirty-seven patients underwent scleral buckling and were included in this study. There were 39 eyes in the buckling group and 35 eyes in the control group (n = 74). Two patients underwent bilateral scleral buckling and both of their eyes were included in the analysis: both patients were bilaterally phakic with one macula off retinal detachment and one macula on retinal detachment. Patients’ age ranged from 31 to 67-years-old (Median: 44). Twenty-four patients (64.8%) were male. Thirty-six of 39 eyes in the scleral buckle group (92.3%) were phakic and 3 eyes (7.7%) were pseudophakic.

Level of inter-rater agreement for measurements of accomodative amplitude were strong (Kappa: 0.83). In phakic eyes, there was a statistically significantly greater difference in accommodative amplitude compared to fellow non-operated eyes at 1 month (0.99 diopters, p value = 0.002) and 3 months (1.17 diopters, p value = 0.001) post-operatively (Tables 1 and 2). Pseudophakic eyes did not show a significant difference in accommodative amplitude when compared to control eyes (p value = 0.86).

**Table 1:** Accommodation amplitude 1 month after scleral buckling surgery in different subgroups

Parameter	Group		P§
	Operated eye AA at 1 m (D)	Control eye AA at 1 m (D)	
Lens status			
Phakic (n = 36) mean ± SD	7.67 ± 4.12	6.68 ± 3.57	0.002
Pseudophakic (n = 3) mean ± SD	5.2 ± 1.31	4.92 ± 2.40	0.86
p	0.25		
Buckle type			
Band & tire (n = 13) mean ± SD	7.27 ± 3.01	6.78 ± 2.94	0.04
Sponse505 (n = 26) mean ± SD	7.34 ± 4.5	6.42 ± 3.87	0.03
p	0.37		
Macula status			
On (n = 10) mean ± SD	7.28 ± 3.4	6.71 ± 3.33	0.46
Off (n = 29) mean ± SD	7.64 ± 4.33	6.48 ± 3.7	0.001
p	0.98		
Age			
≤ 40 (n = 18) Mean ± SD	10.39 ± 3.55	8.65 ± 3.16	0.001
> 40 (n = 21) Mean ± SD	4.84 ± 2.19	4.66 ± 2.76	0.59-
p	< 0.001		
Quadrant			
Inferior (n = 14) mean ± SD	8.89 ± 4.17	7.59 ± 3.73	0.04
Others (n = 25) mean ± SD	7.01 ± 2.29	5.63 ± 4.54	0.16
p	0.14		
Quadrant number			
1 (n = 2) mean ± SD	6.17 ± 5.14	5.13 ± 4.11	0.08
2 (n = 24) mean ± SD	8.26 ± 3.88	7.44 ± 3.49	0.17
4 (n = 13) mean ± SD	7.28 ± 3.01	6.78 ± 2.94	0.04
p	0.09		
BCVA			
≤ 0.3 (n = 17) Mean ± SD	8.41 ± 3.89	7.48 ± 3.9	0.17
> 0.3 (n = 22) mean ± SD	7.04 ± 4.07	6.11 ± 3.37	0.008
p	0.24		
Total mean ± SD	7.47 ± 4.01	6.55 ± 3.55	0.003

*AA* Accommodation amplitude, *BCVA* Best corrected visual acuity based on logMar, *D* Diopter

p§ p value for comparison of AA in operated Vs fellow eye based on Linear GEE

p: p value for comparison of AA between subgroups of same arrow in operated eye based on Linear GEE

**Table 2:** Accommodation amplitude 3 months after scleral buckling surgery in different subgroups

Parameter	Group		p§
	Operated eye AA at 3 m (D)	Control eye AA at 3 m (D)	
Lens status			
Phakic mean ± SD	7.87 ± 4.26	6.61 ± 3.5	0.001
Pseudophakic mean ± SD	5.2 ± 1.31	4.92 ± 3.40	0.86
p	0.2		
Buckle type			
Band & tire mean ± SD	7.87 ± 3.05	6.82 ± 2.98	0.03
Sponse505 mean ± SD	7.48 ± 4.69	6.30 ± 3.85	0.01
p	0.38		
Macula status			
On mean ± SD	7.28 ± 3.4	6.82 ± 2.98	0.46
Off mean ± SD	7.64 ± 4.33	6.48 ± 3.7	0.001
p	0.93		
Age			
≤ 40 mean ± SD	10.58 ± 3.73	8.52 ± 3.16	0.001
> 40 mean ± SD	4.9 ± 2.28	4.64 ± 2.77	0.4-
p	< 0.001		
Quadrant			
Inferior mean ± SD	8.96 ± 4.15	7.32 ± 3.75	0.03
Others mean ± SD	5.63 ± 4.55	6.55 ± 4.54	0.19
p	0.02		
Quadrant number			
1 mean ± SD	6.387 ± 5.57	7.47 ± 4.11	0.9
2 mean ± SD	8.36 ± 3.84	7.22 ± 3.5	0.08
4 mean ± SD	7.84 ± 3.5	6.82 ± 2.98	0.03
p	0.09		
BCVA			
≤ 0.3 mean ± SD	8.65 ± 4.27	7.26 ± 4.27	0.09
> 0.3 mean ± SD	7.12 ± 4.10	6.12 ± 3.39	0.005
p	0.28		
Total mean ± SD	7.6 ± 4.16	6.48 ± 3.54	0.001

AA: Accommodation amplitude. BCVA: Best corrected visual acuity based on logMar. D: Diopter

p§: p value for comparison of AA in operated Vs fellow eye based on Linear GEE

p: p value for comparison of AA between subgroups of same arrow in operated eye based on Linear GEE

Patients who underwent encircling (band or tire) or segmental buckles (sponge) both had a statistically significantly higher accommodative amplitude compared to control eyes at one month (p = 0.04 and p = 0.03, respectively) and three months (p = 0.03 and p = 0.01, respectively). The differences in accommodative amplitude between these two surgical groups did not reach statistical significant at the 1 month (p = 0.37) or 3 months post-operative visit (p = 0.38). Among patients who received segmental sponges, those with inferior placement achieved a greater increase in accommodative amplitude compared to those with elsewhere placement at the 3 month post-operative visit (p value = 0.02).

Buckle placement further from the limbus was associated with less difference in accommodative amplitude compared to control eyes (p = 0.04). Patients with greater BCVA and age under 40 showed a larger difference in accommodative amplitude compared to control eyes (Table 1 and 2).

Patients with age under 40 showed significantly higher postoperative accommodative amplitude compared to more than 40 year old patients (p value < 0.001 for both 1 and 3 months post-operation, see Table 1 and 2).

### Discussion

Efforts to use scleral expansion as a presbyopia treatment have resulted in contradictory subjective and objective outcomes. [1, 7–10] Scleral buckling surgery modifies scleral geometry to reduce traction on retinal breaks by changing the scleral architecture [11]. Scleral buckling can induce a myopic shift and has been shown to alter scleral shape, stress distribution and rigidity. [11–14].

This study demonstrated that eyes undergoing scleral buckling had one diopter more accommodative amplitude after surgery than control eyes. Higher amounts of accommodative amplitude relative to control eyes were present in eyes undergoing encircling buckles and segmental buckles, with the differences between these two surgical approaches not reaching statistical significance. This suggests that even a more localized change in scleral architecture can induce a disproportionate change in the shape of the lens or that only a segmental change in lens steepness is necessary to increase accommodative amplitude. Among eyes with segmental buckles, those with inferior placement had a larger change in accommodative amplitude when compared to control eyes. There was no significant difference in accommodative amplitude in pseudophakic eyes likely because the rigidity of intraocular lenses prevents induced changes in lens shape. Of note, the low number of pseudophakic eyes in this study limits the statistical power of these comparisons.

Buckle distance from the limbus, age, and post-operative BCVA correlated with the magnitude of the difference in accommodative amplitude compared to control eyes. Younger patients have greater accommodative reserve and more pliable phakic lenses which may allow for greater changes in accommodative amplitude after surgery. Buckle placement further from the limbus was associated with less change in accommodative amplitude compared to fellow eyes, possibly because the effect on the zonules and lens is mitigated the further the buckle, and resultant scleral expansion, is from the ciliary body.

Further studies can help explore the long-term changes in accommodative amplitude in patients undergoing scleral buckling. Anatomic studies using ultrasound biomicroscopy, axial length measurements, and pupil size can lend further insights into the mechanisms behind post-operative changes in accommodation. This study is limited by its sample size which can limit the ability to detect small changes between groups. Scleral buckling resulted in modest, but statistically significant, differences in the accommodative amplitude of phakic eyes when compared to control eyes. This information can be helpful in pre-operative planning, patient selection, and counseling in retinal detachment repair. Segmental changes in scleral architecture resulted in comparable relative changes in accommodative amplitude with inferior buckle placement achieving a greater relative difference in accommodative amplitude. Scleral approaches to presbyopia treatment have been gaining attention and these findings may help shed light on how to maximize these interventions.

Measurement of axial length of the globe, anterior chamber depth and patients’ pupil size and identifying their role in increasing accommodation amplitude after scleral buckling surgery have not been done in this study and is suggested in future studies.

The paper has been accepted for oral presentation in 37th World Ophthalmology Congress of the ICO taking place in Cape Town, South Africa.
